# Effect of gentiopicroside on endogenous formaldehyde homocysteine–pathway related proteins in rats with non-alcoholic steatohepatitis

**DOI:** 10.3389/fphar.2025.1700101

**Published:** 2026-01-06

**Authors:** Jiaxin Chen, Huiling Zuo, Yuhang Jiao, Huanhuan Zhao, Xuan Ma, Yahui Xiao, Xiangqiong Li, Wei Zhao, Anhua Shi, Wenhui Chen

**Affiliations:** 1 Yunnan Key Laboratory of Integrated Traditional Chinese and Western Medicine for Chronic Disease in Prevention and Treatment, Yunnan University of Chinese Medicine, Kunming, China; 2 Key Laboratory of Microcosmic Syndrome Differentiation, Yunnan University of Chinese Medicine, Kunming, China; 3 Department of Basic Medical, Yunnan University of Chinese Medicine, Kunming, China; 4 Chongqing Traditional Chinese Medicine Hospital, Chongqing, China

**Keywords:** non-alcoholic steatohepatitis, gentiopicroside, oxidative stress, endogenous formaldehyde, lipid metabolism

## Abstract

**Background:**

Non-alcoholic steatohepatitis (NASH) is a progressive form of non-alcoholic fatty liver disease (NAFLD) characterised by high prevalence, increasing incidence among younger individuals and poor prognosis. NASH pathophysiology is not completely understood, and at present, there are no viable pharmacological treatments for this condition in clinical practice. Gentiopicroside (GPS). Can alleviate NASH by reducing inflammatory responses, inhibiting oxidative stress and influencing blood lipid levels.

**Objective:**

To examine the preventive and therapeutic effects of gentiopicroside (GPS) on proteins and metabolites related to the endogenous formaldehyde-homocysteine (FA-HCY) pathway as well as on oxidative stress in NASH rats.

**Methods:**

A high-fat, high-sugar diet was used to create a NASH rat model, and the rats’ liver weight, body weight and liver index levels were measured. Oil red O and hematoxylin and eosin (HE) stainings were used to detect pathological alterations in the rat livers. Serum biochemical kits were used to identify biochemical markers in the rat serum. Markers linked to oxidative stress and metabolites associated with the endogenous FA-HCY pathway index were identified using Enzyme-linked immunosorbent assays (ELISA) kits. Gas chromatography was employed to measure the amount of endogenous FA in the liver and serum. Western blotting and real-time (RT) PCR were used to determine the relative expression levels of proteins and mRNAs associated with the endogenous FA-HCY pathway in rat liver tissues. Immunofluorescence was used to measure the relative fluorescence intensities of the proteins MTHFR, MAT1A and ALDH2.

**Results:**

A diet rich in fats and sugars results in weight gain and considerable steatosis. GPS can mitigate hepatic steatosis in NASH rats, reduce NAFLD activity scores and decrease the oil red O-stained area. The NASH rats’ serum biochemical markers should be improved. In groups treated with GPS (low, medium and high dosages), the serum exhibited increased HDL-C levels (*P* < 0.05) and considerably lowered the levels of total cholesterol, TG, ALT, AST and LDL-C. According to oxidative stress indicators, GPS increased SOD, GSH, Glutathione S-transferases (GST) and CAT levels (*P* < 0.05) and decreased MDA and reactive oxygen species levels in the blood of NASH rats. Serum pathway indicators demonstrated that GPS also decreased the levels of SAH and HCY in the serum and endogenous FA in the livers of NASH rats (*P* < 0.05) while simultaneously increasing the level of SAM (*P* < 0.05). Compared with the model group, the medium-dose GPS treatment group exhibited elevated endogenous FA levels in the blood (*P* < 0.05). Protein analysis revealed a significant reduction in AHCY expression (*P* < 0.05) but a significant increase in ALDH2 and CBS levels (*P* < 0.05) in the GPS treatment groups. RT PCR results indicated that GPS decreased AHCY and related mRNA expression (*P* < 0.05) while enhancing the levels of MTHFR, MAT1A and ALDH2 and related mRNA expression (*P* < 0.05).

**Conclusion:**

The GPS has therapeutic benefits in NASH rats as it improves liver weight, body weight, liver index levels, liver steatosis and liver function. The efficacy of GPS in ameliorating NASH in rats may be associated with the modulation of the endogenous FA-HCY pathway and attenuation of oxidative stress.

## 1 Introduction

Non-alcoholic steatohepatitis (NASH), development of non-alcoholic fatty liver disease (NAFLD), is characterised by liver steatosis ≥5%, hepatocyte injury, inflammation and various degrees of liver fibrosis (Chalasani et al., 2018). The global frequency of NASH in the general population is 2%–6% (Kadi et al., 2024). The occurrence and development of this condition involve various factors, including insulin resistance (Branković et al., 2022), disturbances in glucose and lipid metabolism (Wang et al., 2022), dietary imbalances and intestinal microbiota (Yang et al., 2023) and genetic polymorphisms (Lee et al., 2022). The pathogenesis of NASH is not completely understood, and there are currently no effective pharmacological treatments available in clinical practice (Wei et al., 2024). Therefore, identifying pharmacological agents to effectively treat NASH is vital.

Endogenous formaldehyde (FA) serves as a methyl donor and participates in numerous cellular biological processes *in vivo*, such as one-carbon metabolism, nucleotide synthesis, DNA methylation, epigenetic modification, gene expression regulation and metabolic maintenance (Burgos-Barragan et al., 2017; Ridpath et al., 2007; Yu et al., 2015). Alcohol dehydrogenase 5 (ADH5) (Morellato et al., 2021) or aldehyde dehydrogenase 2 (ALDH2) (Kim et al., 2020; Nattermann et al., 2023) can convert endogenous FA into formate (Umansky et al., 2022).

It can directly engage in one-carbon metabolism and spontaneously condense with tetrahydrofolate (THF) to yield 5,10-CH_2_-THF. This compound then participates in one-carbon metabolism (Morellato et al., 2021), which encompasses the folic acid and methionine cycles. Homocysteine (HCY) is generated from methionine by removing its terminal methyl group and serves as an intermediary in the methionine cycle (Besen et al., 2024). Dysregulation of methionine metabolism can lead to fat buildup and is intimately associated with the pathological alterations in hepatic steatosis (Murthy et al., 2003; Radziejewska et al., 2020). HCY can undergo trans-sulfuration to yield cysteine, which is further metabolised by Cystathionine -β -synthase (CBS) to generate hydrogen sulphide (H_2_S) and GSH (Dahlhoff et al., 2013). The accumulation of HCY, prevention of its remethylation and trans-sulfuration and inadequate production of GSH to mitigate the effects of reactive oxygen species (ROS) contribute to promoting oxidative stress (Mato et al., 2017; Raghubeer and Matsha, 2021).

Moreover, HCY can directly produce ROS through auto-oxidation or lead to ROS accumulation by disturbing the equilibrium between pro-oxidative and anti-oxidant activities (Harb et al., 2020). HCY build up can enhance lipid peroxidation reactions (Fang et al., 2023), trigger oxidative stress, activate inflammatory and fibrotic signalling pathways (Gonzalez et al., 2020), lead to inflammatory infiltration of hepatocytes (Gulsen et al., 2005) and facilitate the progression of steatosis to NASH (Liu et al., 2015).

In conclusion, endogenous FA build up and the mismatch between supply and demand of one-carbon units result in abnormalities in one-carbon metabolism that cause HCY accumulation, further encouraging lipid build up and oxidative stress and leading to NASH. Consequently, dysfunction of the endogenous FA-HCY pathway can facilitate the onset and progression of NASH.

Gentiopicroside (GPS) is a cyclic ether terpene glycoside extracted from plants of genus *Gentiana* and family Gentianaceae. This molecule exhibits several pharmacological properties, including cholesterol-lowering, anti-inflammatory, anti-oxidant and hepatoprotective properties and plays a crucial role in managing NAFLD by reducing lipid build up (Choubey et al., 2024; Dai et al., 2018; He et al., 2015; Liu et al., 2024; Xiao et al., 2022).

This study replicated the rat model of NASH to observe the preventive and therapeutic effects of GPS on NASH rats, and it preliminarily explored the relationship between the therapeutic effect of GPS and the metabolism-related indicators of the endogenous formaldehyde-homocysteine pathway, as well as oxidative stress.

## 2 Materials and methods

### 2.1 Experimental materials

#### 2.1.1 Experimental animals

In total, 50 male Sprague–Dawley rats, weighing 160–180 g, of SPF grade and aged 6 weeks, were acquired from the Laboratory Animal Center of Kunming Medical University (licence no.: SCXK (Dian) 2020–0004). The experimental animals were housed in individual cages (six animals per cage) at the Animal Experiment Center of Yunnan University of Traditional Chinese Medicine. Temperature was maintained at 22 °C–27 °C, with relative humidity of 40%–60%. The rats had free access to food and water, and periods of 12 h of light and 12 h of darkness were alternated to replicate the typical circadian rhythm. The experimental protocols regarding diet, anaesthesia, blood and tissue sample collection and disposal of dead animals complied with the ethical guidelines issued by the Experimental Animal Ethics Committee of Yunnan University of Traditional Chinese Medicine (No. R-062023223).

#### 2.1.2 Experimental reagents

GPS with a purity ≥98% (Baoji Chenguang Biotechnology Co., LTD., HR2475W19); polyene phosphatidylcholine (Sanofi [Beijing] Pharmaceutical Co., LTD., National Drug Approval No. EBJD4198); mouse Afodin solution (G0-R047, Beijing Hanhai Tuoxin Biotechnology Co., LTD.). Total cholesterol (TC), triglycerides (TG), alanine aminotransferase (ALT), aspartate aminotransferase (AST), high-density lipoprotein cholesterol (HDL-C), low-density lipoprotein cholesterol (LDL-C) kits were purchased from the Nanjing Jiancheng Institute of Bioengineering with item numbers A111-1-1, A110-1-1, C009-2-1, C010-2-1, A112-1-1 and A113-1-1, respectively. ROS, GSH, MDA, SOD, SAH, SAM, GST, CAT, GSH, HCY were obtained from Jiangsu Enzyme Immunoassay Biotechnology Co., LTD. Their item numbers are MM-88564O1, MM-20251R1, MM-2037H1, MM-20387R1, MM-50456H1, MM-0248H1, MM-21254R1, MM-20447R1, MM-20251R1 and MM-50456H1. β-actin antibody (Proteintech, 20536-1-AP); ALDH2 antibody (Proteintech, 15310-1-AP); MAT1A antibody (Proteintech, 12395-1-AP); MTHFR antibody (Proteintech, 26591-1-AP); CBS antibody (Proteintech, 14787-1-AP); AHCY antibody (Proteintech, 10757-2-AP); PVDF membrane with a thickness of 0.45 μm (IPVH00010, Millipore); Universal total RNA extraction reagent (U7431, Suzhou Youyi Landi Biotechnology Co., LTD.) Taq SYBR® Green qPCR Premix (Universal) (EG0117M, Jiangsu Bishi Mei Biotechnology Co., LTD.) All-in-One First-Strand Synthesis MasterMix (with dsDNase) was obtained from Jiangsu Bestmate Biotechnology Co., LTD. (item number (EG15133S).

### 2.2 Experimental methods

#### 2.2.1 Experimental animals

The 50 SPF-grade Sprague–Dawley rats selected for this experiment were acclimatised for 1 week and subsequently randomly assigned to control (n = 8) and modeling (n = 42) groups. The modeling group received a high-fat diet supplemented with 5% brown sugar water for 12 weeks. After the model verification is successful, the Modelin group will be randomly assigned to the model group (n = 8, from the original group), one polyene phosphatidylcholine (PPC) treatment (23.3 mg/kg, n = 8) and three GPS treatment groups at low, medium and high doses. GPS-D (50 mg/kg, n = 8),GPS-Z (100 mg/kg, n = 8),GPS-G (200 mg/kg, n = 8), respectively. Each drug was administered for 4 weeks. The rats were weighed on a weekly basis, and liver and serum samples were obtained at the end of the experiment.

### 2.3 Measurement of indicators

#### 2.3.1 Serum biochemical indicators

The levels of TC,TG, LDL-C,HDL-C, ASTand ALT in the serum of rats in each group were measured using biochemical kits. and calculated liver index. The liver index is as follows: liver mass/body mass × 100%.

#### 2.3.2 Hematoxylin and eosin (H&E) staining method

Liver tissue samples were preserved in 4% paraformaldehyde solution, embedded in paraffin, sectioned into 5-μm thick slices and stained with H&E reagent per standard protocols. Then, histological alterations were examined using slide scanning image analysis (SQS1000-S, Shengqiang Technology Co., LTD.). Steatosis, lobular inflammation and hepatocyte ballooning were assessed to determine the severity of NAFLD using the NAFLD activity score. The specific evaluation criteria are included in Table 1. NAS <3 excludes NASH, 3–4 may indicate NASH and ≥4 confirms the presence of NASH.

**TABLE 1:** Scoring criteria for NAS.

Project	0 points	1 points	2 points	3 points
Hepatocyte steatosis	<5%	5%∼<33%	33%∼<66%	>66%
Inflammation within the lobules	No	<2/200x field of view	2–4/200x field of view	>4/200x field of view
Balloon-like transformation of hepatocytes	No	Occasionally seen	Common	​

Following comprehensive theoretical instruction, two students from our research group performed a blind evaluation of liver tissue sections. Six pathological specimens were selected from each group, for 36 slice samples. The intensities of hepatic inflammation, necrosis and fibrosis were assessed to determine the NAFLD activity score. The mean scores of the two students were computed, and the data were analysed in SPSS v. 27.0.

#### 2.3.3 Liver oil red O staining

The liver lobules preserved in 4% paraformaldehyde will be pruned and dehydrated. After embedding them with OCT adhesive, the samples were frozen, sectioned, dyed with oil red O and sealed with glycerol gelatin. After drying, they were photographed using a glass slide scanner. In this technique, lipid droplets are coloured red, whereas cell nuclei are dark blue. The oil red O-stained area was quantitatively assessed using ImageJ. Oil Red O expression = (area value of lipid droplets in each group/mean total area) × 100%. The acquired data were analysed in SPSS 27.0 and the results were displayed as ( $\bar{x}\pm S$ ).

#### 2.3.4 Enzyme-linked immunosorbent assays (ELISA)

ELISA kits were used to measure the levels of HCY, SAM, SAH, ALDH2, CBS, MDA, SOD, ROS, GSH, GST and CAT expression in rat serum. All procedures were carefully executed by following kit instructions, and preliminary studies were conducted for each assay. Optical density was measured using a multi-functional microplate reader (Synergy LX, BIOTEK, Berten Instruments, USA), and the standard curve equation was calculated based on the concentration of the standard substance. The index concentration was computed, followed by data collection, and analysed using SPSS 27.0. The results were visualised as ( $\bar{x}\pm S$ ).

#### 2.3.5 Gas chromatography-mass spectrometry

Gas chromatography-mass spectrometry was used to determine the amounts of endogenous FA in the rats’ livers and serum. Samples were processed by adding 1% formic acid water and subjecting them to ultrasonic extraction for 30 min. Then, 1 mL of the sample solution was added with 100 μL of 2, 4-dinitrophenylhydrazine solution (10 mg/10 mL 1% formic acid) and placed in a water bath at 60 °C for derivatisation for 30 min. After rapid cooling, 1 mL of n-hexane solution was added, and the mixture was shaken for extraction. Then, the upper layer was removed and loaded into the chromatograph for analysis. The FA standard products were derived simultaneously, with standard concentrations of 10, 20, 50, 100 and 200 μg/L. The following conditions were maintained for operating the chromatograph (Agilent 7890B): chromatographic column, DB-5; detector, ECD; injection port, 250 °C; detector, 300 °C; split ratio, 10:1; gradient, maintain at 80 °C for 1 min, increase from 20 °C/min to 200 °C, then from 30 °C/min to 280 °C and maintain for 2 min.

#### 2.3.6 Western blot analysis

Liver tissue samples, stored in a freezer at −80 °C, were homogenised using radioimmunoassay assay (RIPA) buffer (Beyotime Biotechnology, Shanghai, China). Each sample was separated using a 10% SDS-PAGE gel and subsequently transferred to a PVDF membrane. The membrane was blocked with 5% skim milk for 2 h and subsequently incubated overnight at 4 °C with the ALDH2, MAT1A, MTHFR, CBS and AHCY antibodies. Another incubation step with a suitable HRP-conjugated secondary antibody was then carried out at room temperature for 1 h. ImageJ was used to analyse protein levels, which were normalised based on β-actin levels. The data were analysed in SPSS 27.0, and the results were visualised as ( $\bar{x}\pm S$ ).

#### 2.3.7 Real-time (RT) PCR

Total RNA was isolated from the frozen rat liver tissues using RNA extraction kits, and RNA purity was subsequently assessed using a micro-spectrophotometer. Next, cDNA was synthesised from 1 μg of high-quality total RNA using a reverse transcription premix kit. RT PCR amplification was conducted at the following conditions: pre-denaturation at 95 °C for 30 s, denaturation at 95 °C for 5 s, annealing at 55 °C for 30 s and extension at 72 °C for 30 s. Amplification was conducted for 40 cycles, using β-actin as the internal reference gene, with the 2^−ΔΔCT^ value representing the relative level of mRNA expression. The data were analysed in SPSS 27.0, and the results were visualised as ( $\bar{x}\pm S$ ). The primer sequences are included in Table 2.

**TABLE 2:** The upstream and downstream primer sequences in the experiment.

Primer name	Primer sequence (5′-3′)
ALDH2-F	GGT​TGG​TGG​GCG​GTG​TTT​G
ALDH2-R	GCGTGTGCGTGCGTGTG
MTHFR-F	GCG​TAT​CAG​CCT​TGC​CTT​CAC
MTHFR-R	CTCTGGTCCTGCCTCAAC
CBS-F	GGGATGGTGACTGGGAAC
CBS-R	GGA​TCG​GCT​TGA​ACT​GCT​TGT​AG
AHCY-F	ATA​TCA​TCC​TTG​GTC​GGC​ACT​TTG
AHCY-R	CCT​TCT​CCA​CAG​CGT​TGT​CAT​TG
MAT1A-F	TGC​CGC​TCA​CCA​TTG​TTC​TTG
MAT1A-R	AAT​CAG​GTC​TCA​GCC​AGG​GAA​G
β-actin-F	ACT​GCC​GCA​TCC​TCT​TCC​TC
β-actin-R	GAA​CCG​CTC​ATT​GCC​GAT​AGT​G

#### 2.3.8 Immunofluorescence analysis

Paraffin sections were prepared from the rat liver tissue samples. A dewaxing solution was used for the dewaxing process, and EDTA was employed for repair purposes. After the samples cooled naturally, they were blocked with 3% BSA for 30 min and then supplemented with the primary antibody overnight. The next day, they were washed with PBS three times and incubated with the secondary antibody at room temperature for 50 min. Images were captured via self-fluorescence quenching and then analysed using ImageJ. Mean fluorescence intensity was calculated as the sum of fluorescence intensities within the specified region (IntDen) divided by the area. The data were analysed in SPSS v. 27.0, and the results were visualised as ( $\bar{x}\pm S$ ).

### 2.4 Statistical analysis

All data were analysed in SPSS v. 27.0, and the results were presented as the mean ± standard deviation. Before analysis, tests were conducted to assess the normality of data distribution for each group and the homogeneity of variances. For homogeneous variances, one-way ANOVA was employed for group comparisons, followed by the LSD test, whereas for heterogeneous variances Tamhane’s T2 all-pairs comparison test was conducted instead. Statistically significant differences were defined by a *P*-value of <0.05. Multiple groups of non-normally distributed data were analysed using the non-parametric Kruskal–Wallis test for independent samples, with statistical significance also set at *P*-value of <0.05. Graphs depicting statistical results were plotted using GraphPad Prism v. 10.1.2.

## 3 Results

### 3.1 Effects of GPS on serum lipid metabolites and liver function in NASH rats

**FIGURE 1:**
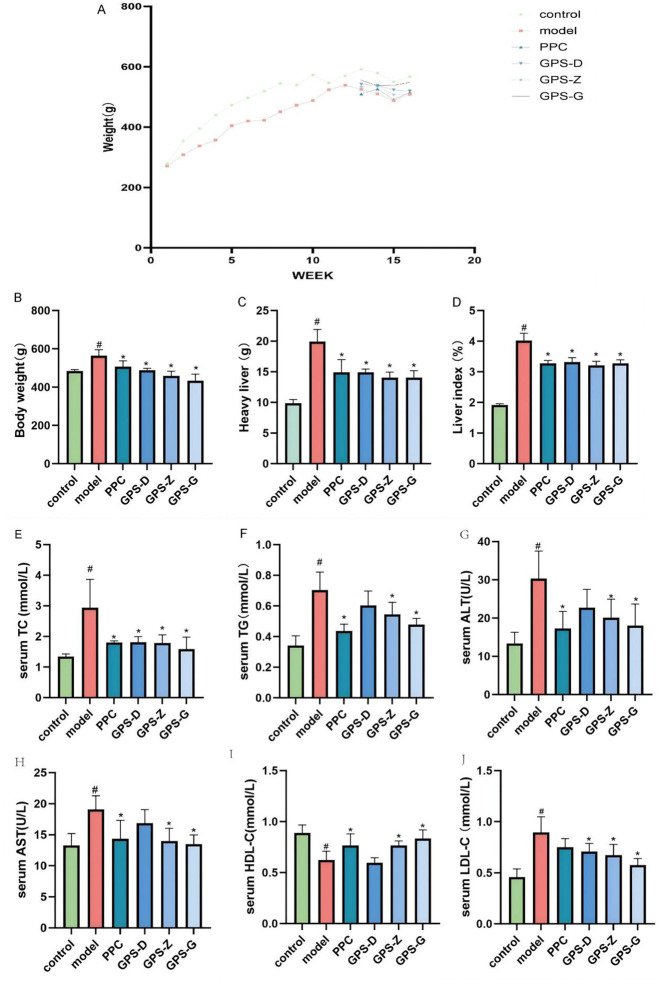
GPS enhances body weight, blood lipid metabolites, and liver function parameters in NASH rats. **(A)** Time-course body weight curves for each group of rats; **(B)** Body weight levels of rats in each group (n = 6); **(C)** Liver weight levels of rats in each group (n = 6); **(D)** Liver index levels of rats in each group (n = 6); **(E)** Serum TC levels of rats in each group (n = 6); **(F)** Serum TG levels of rats in each group (n = 6); **(G)** Serum ALT levels of rats in each group (n = 6); **(H)** Serum AST levels of rats in each group (n = 6); **(I)** Serum HDL-C levels of rats in each group (n = 6); **(J)** Serum LDL-C levels of rats in each group (n = 6). Note: Data are expressed as the mean ± SD. “#” indicates that *P* < 0.05 compared with the normal group; “*” indicates that *P* < 0.05 compared with the model group.

**FIGURE 2:**
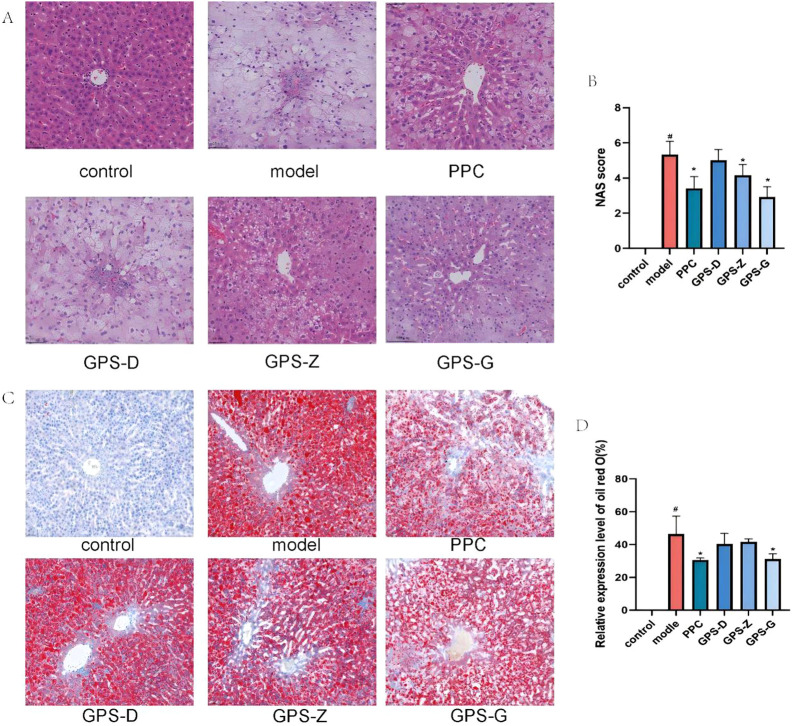
GPS mitigates lipid accumulation and the infiltration of inflammatory factors in the livers of NASH rats. **(A)** H&E staining results of livers in each group of rats (20 × 10); **(B)** NAS score levels of rats in each group (n = 6); **(C)** Results of oil red O staining in each group of rats (scale = 500 μm); **(D)** Expression levels of oil red O area in each group of rats (n = 6). Note: Data are expressed as the mean ± SD. “#” indicates that *P* < 0.05 compared with the normal group; “*” indicates that *P* < 0.05 compared with the model group.

The liver index, defined as the ratio of liver weight to body weight in rats, serves as a notable predictor of hepatic fat build up. The GPS treatment groups exhibited a significant decrease in body and liver weight, and thus in the liver index (*P* < 0.05). Notably, GPS can mitigate NASH in rats. Compared with the control group, the model group had elevated levels of serum TC, TG, AST, ALT and LDL-C (*P* < 0.05). Compared with the model group, the PPC treatment group exhibited elevated levels of TC, TG, ALT, AST and HDL-C (*P* < 0.05) but low levels of LDL-C, although this difference was not statistically significant. All GPS treatment groups (three dosages) exhibited significantly reduced levels of TC, TG, ALT, AST and LDL-C (*P* < 0.05) but increased levels of HDL-C (*P* < 0.05) (Figure 1). Therefore, GPS boosts lipid deposition in the liver in NASH rats, improving its function (Figure 2).

### 3.2 Influence of GPS on liver pathological indicators

The H&E staining results indicated a distinct liver architecture in the control group rats, with hepatocytes arranged in an orderly and compact manner. In contrast, in the model group, the organisation of hepatocytes was aberrant: cells were palely stained and lipid vacuoles along with inflammatory infiltration were present in the cytoplasm. Compared with the model group, the hepatocytes in the GPS-D, GPS-Z, GPS-G and PPC treatment groups exhibited an orderly arrangement, with fewer lipid vacuoles and inflammatory infiltration. The NAFLD activity scores of rats in the model group were significantly higher than those of rats in the control (*P* < 0.05) and GPS-Z, GPS-G and PPC treatment groups (*P* < 0.05).

The result of oil red O staining indicated a distinct liver architecture in the control group, with minimal formation of red lipid droplets. A considerable accumulation of red lipid droplets was observed in the livers of rats in the model group. A notable amelioration of hepatic steatosis was detected in the GPS-D, GPS-Z, GPS-G and PPC treatment groups. The relative expression level of oil red O was significantly higher in the model group than in the control (*P* < 0.05) and GPS-G and PPC treatment groups (*P* < 0.05). For the GPS-Z and GPS-D groups, the relative expression levels of oil red O were also lower than those in the model group; however, the differences were not statistically significant (Figure 2).

### 3.3 Effects of GPS on serum oxidative stress levels and metabolites related to the endogenous FA-HCY pathway in NASH rats

**FIGURE 3:**
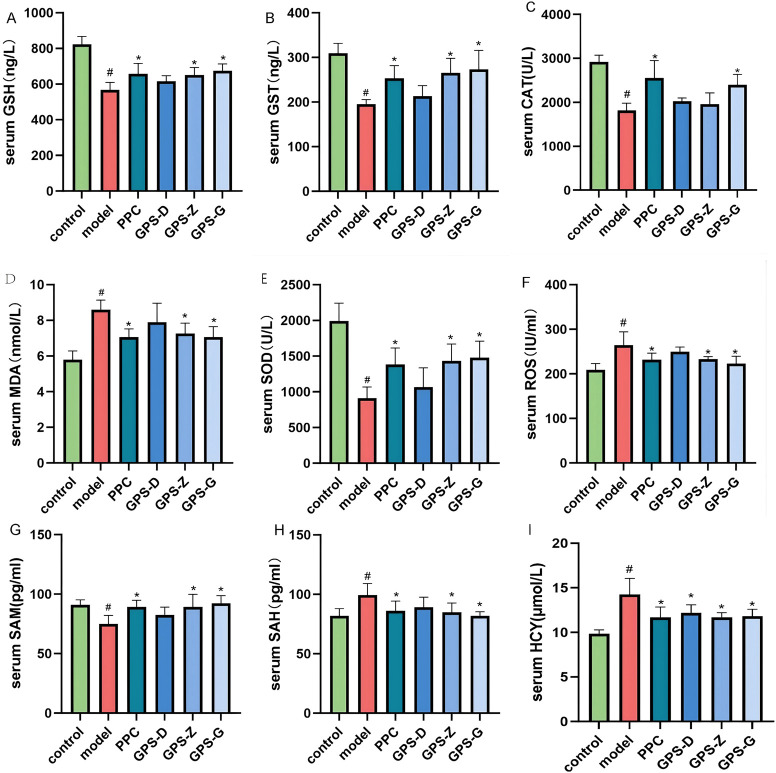
In NASH rats, GPS lowers serum oxidative stress levels. **(A)** Serum GSH levels of rats in each group (n = 6); **(B)** Serum GST levels of rats in each group (n = 6); **(C)** Serum CAT levels of rats in each group (n = 6); **(D)** Serum MAD levels of rats in each group (n = 6); **(E)** Serum SDO levels of rats in each group (n = 6); **(F)** Serum ROS levels of rats in each group (n = 6); **(G)** Serum SAM levels of rats in each group (n = 6); **(H)** Serum SAH levels of rats in each group (n = 6); **(I)** Serum HCY levels of rats in each group (n = 6). Note: Data are expressed as the mean ± SD. “#” indicates that *P* < 0.05 compared with the normal group; “*” indicates that *P* < 0.05 compared with the model group.

The model group exhibited higher levels of MDA and ROS (P < 0.05) but lower levels of SOD, GSH, GST and CAT (*P* < 0.05) compared with the control and GPS treatment groups. Considerably, in NASH rats, GPS may lower the degree of oxidative stress in the serum (Figure 3).

The model group showed higher levels of SAH and HCY (*P* < 0.05) but lower levels of SAM, ALDH2 and CBS (*P* < 0.05) compared with the control and GPS-G treatment group (Figure 3; Supplementary Figure S1).

Therefore, GPS may influence markers of metabolites associated with the endogenous FA-HCY pathway in rats with NASH induced by a high-fat and high-sugar diet.

Gas chromatography results indicated that, compared with the control group, the model group rats exhibited a higher level of endogenous FA expression in the liver (*P* < 0.05) but a lower level in the serum, although this difference was not statistically significant. After GPS treatment, the levels of endogenous FA in the serum increased; however, the difference was not statistically significant (Supplementary Figure S1).

### 3.4 Influence of GPS on the expression levels of endogenous FA-HCY pathway-related proteins and mRNA in the livers of NASH rats

**FIGURE 4:**
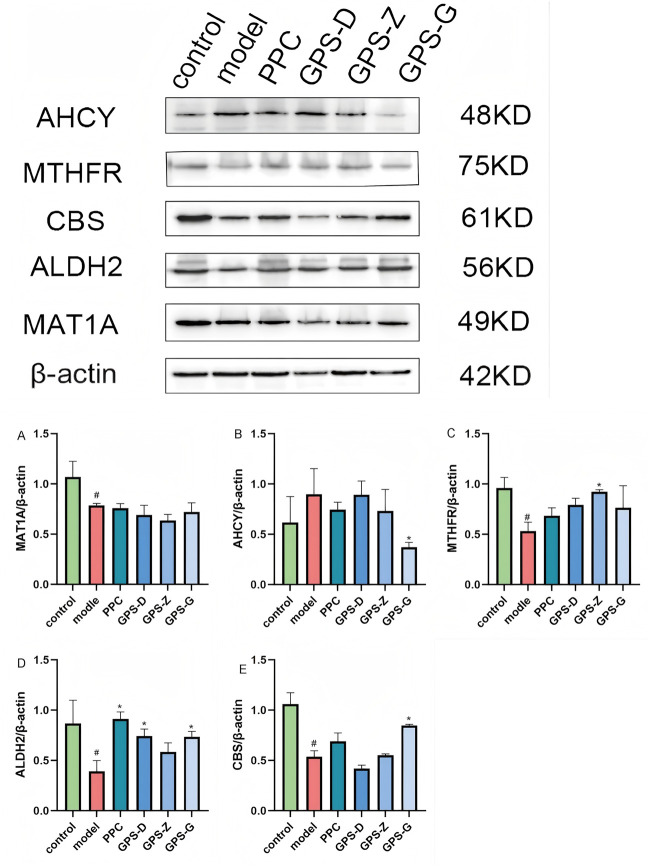
Expression levels of CBS, ALDH2, AHCY, MAT1A and MTHFR proteins in liver tissues of rats in each group. **(A)** Expression levels of MAT1A protein in liver tissues of rats in each group (n = 3); **(B)** Expression levels of AHCY protein in liver tissues of rats in each group (n = 3); **(C)** Expression levels of MYHFR protein in liver tissues of rats in each group (n = 3); **(D)** Expression levels of ALDH2 protein in liver tissues of rats in each group (n = 3); **(E)** Expression levels of CBS protein in liver tissues of rats in each group (n = 3). Note: Data are expressed as the mean ± SD. “#” indicates that *P* < 0.05 compared with the normal group; “*” indicates that *P* < 0.05 compared with the model group.

Western blotting was used to detect the expression of proteins related to the endogenous FA-HCY pathway in NASH rats. Notably, the levels of MAT1A, MTHFR, ALDH2 and CBS expression in the liver in the model group were significantly lower than those in the control group (*P* < 0.05), whereas the level of AHCY expression was higher, although this difference was not statistically significant. Following GPS treatment, the level of AHCY expression decreased (*P* < 0.05). MAT1A levels also decreased, although this change was not statistically significant. Conversely, the levels of ALDH2 and CBS expression increased significantly after GPS treatment (*P* < 0.05). Additionally, an increase was observed for MTHFR expression, but it was not statistically significant (Figure 4).

**FIGURE 5:**
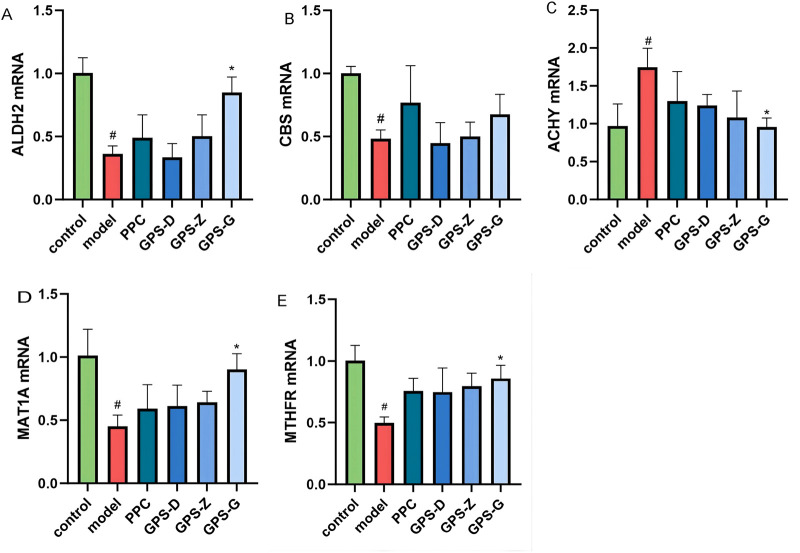
Expression levels of CBS, ALDH2, AHCY, MAT1A and MTHFRmRNA in liver tissues of rats in each group **(A)** Expression levels of ALDH2 mRNA in liver tissues of rats in each group (n = 3); **(B)** Expression levels of MTHFRmRNA in liver tissues of rats in each group (n = 3); **(C)** Expression levels of CBS mRNA in liver tissues of rats in each group (n = 3); **(D)** Expression levels of AHCY mRNA in liver tissues of rats in each group (n = 3); **(E)** Expression levels of MAT1A mRNA in liver tissues of rats in each group (n = 3). Note: “#” indicates that *P* < 0.05 compared with the normal group; “*” indicates that *P* < 0.05 compared with the model group.

The results of RT PCR revealed higher levels of AHCY and related mRNA expression in the liver in the model group than in the control group (*P* < 0.05), but decreased expression of MTHFR, MAT1A, CBS and ALDH2 and related mRNA (*P* < 0.05). The GPS treatment groups exhibited a significant reduction in AHCY and related mRNA expression (*P* < 0.05), along with significant increases in MTHFR, MAT1A and ALDH2 mRNA expressions (*P* < 0.05). CBS and related mRNA expression also increased, although not significantly (Figure 5).

### 3.5 Effect of GPS on the relative fluorescence intensity of endogenous FA-HCY pathway-related proteins

**FIGURE 6:**
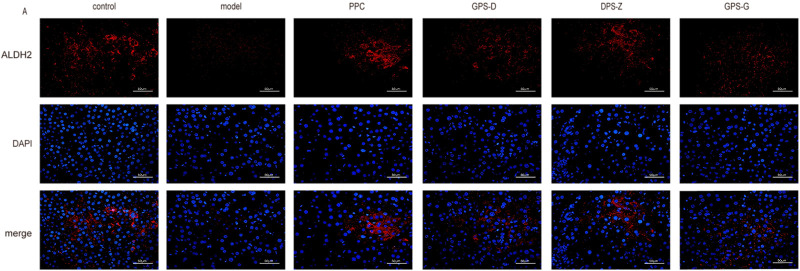
Fluorescence expression intensity of ALDH2 in liver tissues of rats in each group. Scale = 50 μm.

In the fluorescence microscopy images, the proteins ALDH2, MAT1A and MTHFR exhibited red fluorescence, whereas cell nuclei displayed blue fluorescence. The observed fluorescence intensity facilitated the localisation and semi-quantitative analysis of the proteins under investigation. The results showed significantly lower (*P* < 0.05) levels of MTHFR, MAT1A and ALDH2 expression in the liver in the model group than in the control group. Conversely, the expression of these proteins was significantly increased in the GPS treatment groups (*P* < 0.05) (Figure 6; Supplementary Figure S3).

## 4 Discussion

Lipid metabolism disorder is a pathological mechanism of NASH (Ji et al., 2023). Abnormal lipid metabolism results in altered serum levels of TG, TC, HDL-C, and LDL-C (Hegazy et al., 2020). HDL-C and LDL-C serve as primary risk factors for lipid deposition, with a notable positive correlation observed between LDL and liver inflammation (Higashi, 2023). ALT and AST levels correlate with liver function and serve as biomarkers for liver cell inflammatory injury in clinical settings (Chinnappan et al., 2023). Furthermore, ALT is significantly associated with the extent of liver fat accumulation, helping predict fibrosis levels in NASH (Xuan et al., 2024; Zeng et al., 2022).

The experimental results indicated that, in comparison to the control group, the model group rats exhibited increased levels of TC, TG, AST, ALT, and LDL-C, while HDL-C levels decreased. In comparison to the model group rats, the GPS treatment group exhibited decreased levels of TC, TG, AST, ALT, and LDL-C, while HDL-C levels increased. Therefore, GPS enhances liver function and ameliorate lipid metabolism disorders in NASH rats, thereby reducing lipid deposition, similar to the existing literature. For instance (Kou et al., 2023), demonstrated that GPS administration significantly reduced liver function indicators (ALT and AST) in H_2_O_2_-induced HepG2 cells, and two other studies on NASH mice reported that GPS lowered the levels of TC, TG, ALT and AST (Yong et al., 2024; Huang et al., 2024) the levels of TC, TG and LDL-C (Huang et al., 2024) in the serum.

Steatosis and hepatocyte ballooning are the hallmarks of steatohepatitis, a condition characterised by enlarged hepatocytes with a thin or wispy cytoplasm. H&E-stained images allow the quantification and spatial localisation of hepatic steatosis (Mairinoja et al., 2023), which visually appears as a ‘spiderweb’ (Lai et al., 2022). Herein, the H&E-stained images of rat liver tissue samples from each GPS treatment group showed well-aligned hepatocytes, diminished fat vacuoles and inflammatory infiltrates and lower NAFLD activity scores relative to the images obtained for the model group. The integration of H&E and oil red O facilitated a more accurate quantification of hepatic steatosis (expressed as a percentage), enhancing the visibility of lipid droplets (Riva et al., 2018). Oil red O staining indicated reduced area of red lipid droplets in the liver along with decreased relative expression of oil red O in the GPS-treated groups. The hepatic index indicates hepatic lipid deposition (Koehler et al., 2013). Our experimental results demonstrated that, compared with the model group, body weight, liver weight and the hepatic index were significantly reduced across all GPS treatment groups. Thus, GPS effectively reduces liver steatosis and lipid deposition induced by a high-fat and high-sugar diet in NASH rats, while attenuating the degree of pathological changes in the liver.

Polyenephosphatidylcholine (PPC) is a commonly used hepatoprotective agent in clinical practice (Xu et al., 2022), demonstrating favorable therapeutic effects for various liver diseases such as drug-induced liver injury (Li et al., 2022), and non-alcoholic fatty liver disease (Lu et al., 2022; Yang, et al., 2023). PPC is the primary active component of essential phospholipids, playing a crucial role in maintaining cellular membrane fluidity and preserving membrane integrity. It exhibits high bioavailability and multiple biological functions, including inhibiting abnormal lipid accumulation, anti-inflammatory effects (Chen X. et al., 2024) antioxidant properties (Lei et al., 2024), and immunomodulation (Yin et al., 2023). It promotes hepatocyte regeneration and maintains hepatocyte membrane stability, playing a significant role in improving NASH (Lei et al., 2024). In 2000, PPC had already garnered attention for its protective effects against liver damage (Lu et al., 2022; Yang, et al., 2023). PPC can improve and repair damaged hepatocyte membranes. Supplementation with PPC reduces liver weight and improves insulin resistance in mice fed a high-fat diet (HFD). Studies have demonstrated that PPC treatment significantly improves hepatic steatosis (Y. Li et al., 2022) and liver function markers (ALT, AST) (Shao et al., 2025). The objective of this experiment is to validate the effects of GPS on proteins associated with the endogenous formaldehyde-homocysteine pathway in NASH rats. Currently, no targeted drugs exist for this pathway; therefore, PPC, which has demonstrated efficacy against NASH, was selected as the positive control drug.

Oxidative stress directly damages lipids, proteins and DNA, while activating inflammatory and fibrotic signalling pathways (Gonzalez et al., 2020), and it is a crucial factor in the progression of steatosis to NASH. This process is primarily characterised by ROS overproduction, direct depletion of anti-oxidant molecules such as GSH and inhibition of superoxide dismutase (SOD) activity (Liu et al., 2015). GSH is predominantly found in the liver and serves as the primary protector against oxidative stress. ROS can be converted into hydrogen peroxide (H_2_O_2_) through SOD, which is subsequently reduced to H_2_O by GSH, thus safeguarding cells from the detrimental effects of free radicals and pro-oxidants (Averill-Bates, 2023; Gao et al., 2022; Vairetti et al., 2021). ROS can interact with biomolecules, including proteins, leading to lipid peroxidation and the production of malondialdehyde (MDA) (Martín-Fernández et al., 2022). MDA, the end product of lipid peroxidation, significantly contributes to the progression of steatosis to NASH (Cordiano et al., 2023). SOD catalyses the conversion of superoxide anion (•O_2_^−^) into O_2_ and H_2_O_2_, which are subsequently degraded to H_2_O by CAT for metabolic processes (Huang et al., 2021). Glutathione S-transferases (GSTs) are categorised as phase II detoxification enzymes. GSH can bind with various endogenous and exogenous electrophilic compounds (Prysyazhnyuk et al., 2021). GSTs can employ GSH to transform lipid peroxides and other peroxides into less harmful compounds for detoxification (Csiszár et al., 2016). Reportedly, compared with the model group, the GPS treatment groups exhibited decreased levels of MDA and ROS, along with increased levels of SOD, GSH, GST and CAT. Thus, GPS ameliorates serum oxidative stress levels in NASH rats.

The body metabolism of endogenous FA is mostly regulated by the liver. The body can eliminate endogenous FA by metabolising it in the liver and releasing it into the serum. The experimental results of this study indicated that the level of endogenous FA in the liver was higher in the model group than in the control and all three GPS treatment groups. In contrast, endogenous FA content in the serum was lower in the model group than in the control group; however, this difference was not statistically significant. The endogenous FA concentration in the serum in the GPS-G treatment group was higher than that in the model group. We hypothesize that the observed phenomenon may result from increased endogenous formaldehyde levels in the serum due to hepatic metabolism following medication delivery. Currently, there are no direct methods available for the detection of endogenous formaldehyde. The quantities of various metabolites of endogenous formaldehyde can be ascertained by detection. This effort initially intended to utilize high-performance liquid chromatography for the detection of endogenous formaldehyde, given that formaldehyde can be metabolized via many pathways and that metabolite measurements may contain inaccuracies. Nevertheless, in later practice, the concentration of formaldehyde was insufficient for detection. Therefore, gas chromatography-mass spectrometry was selected for the identification of endogenous formaldehyde. Formaldehyde’s tendency to volatilize outside the body may result in losses during transportation and sample processing. Furthermore, the samples may exhibit issues such as significant variability and limited sample sizes attributable to individual differences among rats. Consequently, the statistical differences lack sufficient significance. The effect of a high-fat diet on endogenous formaldehyde concentrations in the body is now ambiguous, necessitating additional experimental validation.

Various methionine metabolites, including SAM, SAH and HCY, are crucial factors influencing lipid levels in the liver. HCY is produced at a crucial juncture in one-carbon metabolism, and blood HCY levels are positively associated with NASH (Matthews et al., 1998; Tripathi et al., 2022). SAH and HCY levels in the serum are significantly correlated with the extent of hepatic steatosis (Tang et al., 2022). Increased SAH levels in particular are a defining feature of NASH lesions (Pogribny et al., 2017). SAM is a principal methyl donor in numerous transmethylation processes and a multi-functional molecule integral to the regulation of hepatic lipid metabolism (Fernández-Ramos et al., 2022). The decrease of SAM levels is a significant catalyst for the onset of human NAFLD and subsequent progression to NASH (Zubiete-Franco et al., 2016). Supplementation with SAM or increasing cysteine intake can enhance GSH synthesis, restore ROS levels, boost TG metabolism in the liver, avoid endoplasmic reticulum stress, minimise *de novo* fat creation in hepatic cells and mitigate liver fat build up (Chen G. et al., 2024; Chen et al., 2023; Van der Crabben et al., 2008). Herein, the GPS treatment groups exhibited higher SAM levels than the model group, but lower levels of SAH and HCY (Bacil et al., 2023). revealed that the livers of mice with NASH induced by a diet rich in saturated fats and sugars exhibited reduced SAH levels and increased SAM levels. Additionally, Tang et al. (2022) indicated that the levels of SAM, SAH and HCY in the serum were remarkably increased in patients with NAFLD.

Given that these disparities may stem from varying diets, the concentrations of methionine metabolites among patients on a methionine-choline deficient, high-fat, and/or standard diet could be discrepant. CBS, the rate-limiting enzyme in the trans-sulfuration pathway, is crucial for detoxifying harmful HCY (Robert et al., 2005). The overexpression of CBS reportedly decreases the plasma HCY levels in mice (Wang et al., 2004). CBS deficiency can lead to hepatic steatosis, inflammation and fibrosis in animal models; nevertheless, the correlation between CBS and NASH remains undetermined (Robert et al., 2005). This study demonstrated that GPS treatment elevated CBS protein and mRNA expression levels in the liver of rats in the mod group; however, the difference in protein expression was not statistically significant. The absence of statistical significance in our observations can be ascribed to the regulation of CBS metabolism by SAM. Specifically, SAM can non-covalently interact with the heme groups of CBS and modulate its redox sensitivity. Thus, reduced SAM concentrations can diminish CBS activity (Werge et al., 2021). Nevertheless, limited studies have been conducted on the potential role of CBS and underlying mechanisms in NASH. Additional tests are required to ascertain whether the transcriptional level of CBS is influenced by other variables. ALDH2, a mitochondrial aldehyde dehydrogenase, is mostly expressed in the liver and scavenges aldehydes *in vivo*, thereby safeguarding cells against oxidative stress damage (Bae et al., 2017; Li et al., 2013; Schug, 2018). By conducting cellular tests with ALDH2 deletion, Bae (Bae et al., 2017) revealed that the absence of aldehyde dehydrogenases may increase the levels of ROS and hazardous aldehydes, further promoting apoptosis (Li et al., 2023). found that NASH worsened in ALDH2 knockout mice, with the lack of this enzyme possibly exacerbating inflammation, facilitating the degradation of the gut flora and inhibiting the FXR pathway. ALDH2 activators such as Alda-1 may mitigate liver damage in mice by improving aldehyde elimination and reversing liver steatosis and apoptosis.

MTHFR is essential for single-carbon metabolism, involving the methionine and folic acid cycles, synthesis of proteins, DNA and RNA, and remethylation of HCY, and is vital for cellular homeostasis. This protein is responsible for regulating the equilibrium of methionine and HCY, averting cellular malfunction and is essential for the methylation of HCY to methionine (Cai et al., 2024; Liew and Gupta, 2015; Raghubeer and Matsha, 2021). Reduced MTHFR and SAM levels and increased SAH levels in the liver in addition to mild micro-alveolar hepatic steatosis reportedly influence the inflammatory and lipid pathways in a murine model (Raghubeer and Matsha, 2021). Our experimental results showed lower levels of MTHFR and mRNA expression in the liver in the model group than in the control and GPS treatment groups. Previous research indicated a potential association between allelic variants of MTHFR (A1298C and C677T) and susceptibility to NAFLD (Hao et al., 2021). Nonetheless, the role of MTHFR polymorphism in the manifestation of NAFLD remains undetermined research across several groups is yet to clarify the influence of MTHFR polymorphism on NAFLD development (An et al., 2017). Liew et al. (Liew and Gupta, 2015) suggest that the MTHFR C677T polymorphism may not be linked to the development of non-alcoholic steatosis into NASH. Consequently, more experiments are required to elucidate these dynamics.

The MAT protein comprises two subtypes, MAT1A and MAT2A, which are predominantly expressed in the adult liver, regulate SAM homeostasis and play a crucial role in lipid metabolism (Sáenz de Urturi et al., 2022). MAT1A can be activated to produce elevated SAM concentrations, establishing a positive feedback loop (Murray et al., 2019). The lack of MAT1A may result in spontaneous steatosis, further progressing to NASH and fibrosis (Fernández-Ramos et al., 2022). Reduced MAT1A expression and diminished SAM levels can induce genomic instability and exacerbate the dysregulation of one-carbon metabolism via many mechanisms during hepatic SAM depletion (Ramani and Lu, 2017). Reportedly, MAT1A^−/−^ mice can spontaneously develop steatosis, exhibiting reduced SOD activity and heightened lipid peroxidation, thereby accelerating the progression of NAFLD to NASH (Robinson et al., 2023). Moreover, mice lacking the MAT1A gene demonstrated reduced SAM/SAH levels and manifested spontaneous NASH at 9–10 months of age (Alarcón-Vila et al., 2023). The experimental results of this study showed lower levels of MAT1A and mRNA expression in the liver in the model group than in the control GPS treatment groups. In contrast, Quinn C et al. (Quinn et al., 2022) reported a substantial upregulation of MAT1A in NASH mice. This discrepancy may be attributed to the body’s compensating mechanisms, along with inherent individual variances among rats. S-adenosylmethionine (AdoMet) can modulate the expression of MAT1A and MAT2A (GarcÍa-Trevijano et al., 2000). The production of AdoMet from various sources may result in fluctuations in the levels of MAT1A. Nevertheless, there is a paucity of studies regarding the potential role of MAT1A and its regulation in NASH. For example, additional tests are required to ascertain whether the transcriptional level of this protein is influenced by other factors.

AHCY is a distinctive enzyme and one of the most conserved proteins across animal groups, crucial for regulating SAM and SAH levels (Krushkal et al., 2016). In mammals, AHCY is the sole enzyme capable of facilitating this process (Vizán et al., 2021). A reduction in AHCY activity will increase SAH levels, hence inhibiting several methylation processes (Matthews et al., 2009). The methylation of AHCY may influence enzyme activity, resulting in abnormalities in HCY metabolism (Li et al., 2020). The suppression of AHCY expression during NASH progression may disrupt trans-sulfuration and transmethylation processes, exacerbating NASH and creating a detrimental feedback loop (Zhang et al., 2020).

**FIGURE 7:**
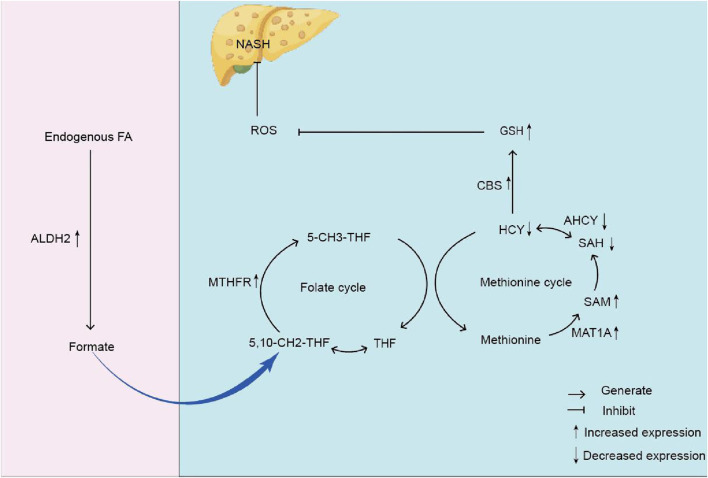
The mechanism diagram of endogenous formaldehyde participating in one-carbon metabolism leading to homocysteine accumulation and causing NASH. Note: formaldehyde (Endogenous FA); Glutathione (GSH) Mitochondrial aldehyde dehydrogenase 2 (ALDH2); Formate; Tetrahydrofolate (THF); 5, 10-methylenetetrahydrofolate (5, 10-CH2-THF); Methylenetetrahydrofolate reductase (MTHFR); Homocysteine (HCY); S-adenosine homocysteine (SAH); Reactive oxygen species (ROS); Non-alcoholic steatohepatitis (NASH); Methionine Cystathionine -β -synthase (CBS); 5-methyltetrahydrofolate (5-CH3-THF); S-adenosylmethionine (SAM); Methionine adenosyltransferase 1A (MAT1A); S-adenosine homocysteine hydrolase (AHCY).

The experiment’s results demonstrated that, in comparison to the control group rats, the model group rats had elevated expression levels of AHCY protein and mRNA in the liver. In comparison to the model group of rats, the expression levels of AHCY protein and mRNA in the GPS therapy group were diminished. Quinn C (Quinn et al., 2022). discovered that AHCY expression was diminished in the livers of NASH mice. Our findings align with those of prior research. However, it must be said that the fundamental mechanism associated with AHCY and NASH is still ambiguous and necessitates additional experimental validation (Figure 7).

The translation of mRNA is a crucial step in the expression of protein-coding genes (Kusnadi et al., 2022). This study reveals that mRNA expression levels of target genes exhibit consistent trends with their corresponding protein expression levels. This finding reflects the synergistic regulatory mechanisms at the transcription-translation interface within this pathway. Despite fluctuations in gene mRNA levels, complex coordination exists between temporally and spatially distinct phases of transcription and translation, maintaining stable levels of specific protein subpopulations through multiple mechanisms (Famà et al., 2025). Furthermore, the effects of high-fat diets on the genes encoding these metabolic enzymes remain poorly understood. Disruption of these enzymes would impair one-carbon metabolism, leading to homocysteine accumulation and promoting the development of NASH. The impact of high-fat diets on endogenous formaldehyde levels remains unclear. Consequently, experimental studies may exhibit elevated methionine metabolism enzymes due to the inclusion of normal dietary methionine content.

In conclusion, GPS ameliorates liver steatosis generated by a high-fat and high-sugar diet in a rat model of NAFLD possibly by affecting the endogenous FA-HCY pathway. Consequently, our research offers a viable therapeutic approach for managing hepatic steatosis and related damage.

## Data Availability

The original contributions presented in the study are included in the article/Supplementary Material, further inquiries can be directed to the corresponding authors.

## References

[B1] Alarcón-Vila C. Insausti-Urkia N. Torres S. Segalés-Rovira P. de la Rosa L. C. Nuñez S. (2023). Dietary and genetic disruption of hepatic methionine metabolism induce acid sphingomyelinase to promote steatohepatitis. Redox Biol. 59, 102596. 10.1016/j.redox.2022.102596 36610223 PMC9827379

[B2] An X. Yang Z. An Z. (2017). MiR-149 compromises the reactions of liver cells to fatty acid *via* its polymorphism and increases non-alcoholic fatty liver disease (NAFLD) risk by targeting methylene tetrahydrofolate reductase (MTHFR). Med. Sci. Monit. Int. Med. J. Exp. Clin. Res. 23, 2299–2307. 10.12659/msm.901377 28507283 PMC5443364

[B3] Averill-Bates D. A. (2023). The antioxidant glutathione. Vitamins Hormones (Vol. 121, pp. 109–141. 10.1016/bs.vh.2022.09.002 36707132

[B4] Bacil G. P. Romualdo G. R. Piagge P. M. Cardoso D. R. Vinken M. Cogliati B. (2023). Unraveling hepatic metabolomic profiles and morphological outcomes in a hybrid model of NASH in different mouse strains. Antioxidants 12 (2), 290. 10.3390/antiox12020290 36829849 PMC9952348

[B5] Bae S. Chon J. Field M. S. Stover P. J. (2017). Alcohol dehydrogenase 5 is a source of formate for *de novo* purine biosynthesis in HepG2 cells. J. Nutr. 147 (4), 499–505. 10.3945/jn.116.244467 28228507 PMC5368588

[B6] Besen S. Ozkale Y. Ceylaner S. Noyan A. Erol I. (2024). Clinical and laboratory findings and etiologies of genetic homocystinemia: a single-center experience. Acta Neurol. Belg. 124 (1), 213–222. 10.1007/s13760-023-02356-1 37728847

[B7] Branković M. Jovanović I. Dukić M. Radonjić T. Oprić S. Klašnja S. (2022). Lipotoxicity as the leading cause zof non-alcoholic steatohepatitis. Int. Journal Molecular Sciences 23 (9), 5146. 10.3390/ijms23095146 35563534 PMC9105530

[B8] Burgos-Barragan G. Wit N. Meiser J. Dingler F. A. Pietzke M. Mulderrig L. (2017). Mammals divert endogenous genotoxic formaldehyde into one-carbon metabolism. Nature 548 (7669), 549–554. 10.1038/nature23481 28813411 PMC5714256

[B9] Cai Y. Liu B. Zhang Y. Zhou Y. (2024). MTHFR gene polymorphisms in diabetes mellitus. Clin. Chim. Acta 561, 119825. 10.1016/j.cca.2024.119825 38908773

[B10] Chalasani N. Younossi Z. Lavine J. E. Charlton M. Cusi K. Rinella M. (2018). The diagnosis and management of nonalcoholic fatty liver disease: practice guidance from the American association for the study of liver diseases. Hepatology 67 (1), 328–357. 10.1002/hep.29367 28714183

[B11] Chen J. Cui X. Fang N. Wu Y. Yu S. Xiao D. (2023). Retracted: methionine-cbs axis promotes intracellular ROS levels by reprogramming serine metabolism. FASEB J. 37 (12), e23268. 10.1096/fj.202300804RRRR 37889798

[B12] Chen G. Zhou G. Zhai L. Bao X. Tiwari N. Li J. (2024). SHMT2 reduces fatty liver but is necessary for liver inflammation and fibrosis in mice. Commun. Biology 7 (1), 173. 10.1038/s42003-024-05861-y 38347107 PMC10861579

[B13] Chen X. Du J. Zhan W. Shao B. Jiang H. Chen Z. (2024). Polyene phosphatidylcholine promotes tibial fracture healing in rats by stimulating angiogenesis dominated by the VEGFA/VEGFR2 signaling pathway. Biochem. Biophysical Res. Commun. 719, 150100. 10.1016/j.bbrc.2024.150100 38763043

[B14] Chinnappan R. Mir T. A. Alsalameh S. Makhzoum T. Adeeb S. Al-Kattan K. (2023). Aptasensors are conjectured as promising ALT and AST diagnostic tools for the early diagnosis of acute liver injury. Life 13 (6), 1273. 10.3390/life13061273 37374056 PMC10305476

[B15] Choubey P. Sharma V. Joshi R. Upadhyaya A. Kumar D. Patial V. (2024). Hydroethanolic extract of Gentiana kurroo Royle rhizome ameliorates ethanol-induced liver injury by reducing oxidative stress, inflammation and fibrogenesis in rats. J. Ethnopharmacol. 325, 117866. 10.1016/j.jep.2024.117866 38350504

[B16] Cordiano R. Di Gioacchino M. Mangifesta R. Panzera C. Gangemi S. Minciullo P. L. (2023). Malondialdehyde as a potential oxidative stress marker for allergy-oriented diseases: an update. Molecules 28 (16), 5979. 10.3390/molecules28165979 37630231 PMC10457993

[B17] Csiszár J. Horváth E. Bela K. Gallé Á. (2016). “Glutathione-related enzyme system: glutathione reductase (GR), glutathione transferases (GSTs) and glutathione peroxidases (GPXs),” in Redox state as a central regulator of plant-cell stress responses (Springer), 137–158.

[B18] Dahlhoff C. Desmarchelier C. Sailer M. Fürst R. W. Haag A. Ulbrich S. E. (2013). Hepatic methionine homeostasis is conserved in C57BL/6N mice on high-fat diet despite major changes in hepatic one-carbon metabolism. PLoS One 8 (3), e57387. 10.1371/journal.pone.0057387 23472083 PMC3589430

[B19] Dai K. Yi X.-J. Huang X.-J. Muhammad A. Li M. Li J. (2018). Hepatoprotective activity of iridoids, seco-iridoids and analog glycosides from gentianaceae on HepG2 cells *via* CYP3A4 induction and mitochondrial pathway. Food Function 9 (5), 2673–2683. 10.1039/c8fo00168e 29675530

[B20] Famà V. Coscujuela Tarrero L. Albanese R. Calviello L. Biffo S. Pelizzola M. (2025). Coupling mechanisms coordinating mRNA translation with stages of the mRNA lifecycle. RNA Biology 22 (1), 1–12. 10.1080/15476286.2025.2483001 40116043 PMC11934187

[B21] Fang W. Jiang L. Zhu Y. Yang S. Qiu H. Cheng J. (2023). Methionine restriction constrains lipoylation and activates mitochondria for nitrogenic synthesis of amino acids. Nat. Communications 14 (1), 2504. 10.1038/s41467-023-38289-9 37130856 PMC10154411

[B22] Fernández-Ramos D. Lopitz-Otsoa F. Millet O. Alonso C. Lu S. C. Mato J. M. (2022). One carbon metabolism and S-adenosylmethionine in non-alcoholic fatty liver disease pathogenesis and subtypes. Livers 2 (4), 243–257. 10.3390/livers2040020 37123053 PMC10137169

[B23] Gao W. Xu B. Zhang Y. Liu S. Duan Z. Chen Y. (2022). Baicalin attenuates oxidative stress in a tissue-engineered liver model of NAFLD by scavenging reactive oxygen species. Nutrients 14 (3), 541–550. 10.1111/1753-0407.13302 35276900 PMC8840060

[B24] GarcÍa-Trevijano E. R. Latasa M. U. Carretero M. V. Berasain C. Mato J. M. Avila M. A. (2000). S‐adenosylmethionine regulates MAT1A and MAT2A gene expression in cultured rat hepatocytes: a new role for s‐adenosylmethionine in the maintenance of the differentiated status of the liver. FASEB J. 14 (15), 2511–2518. 10.1096/fj.00-0121com 11099469

[B25] Gonzalez A. Huerta-Salgado C. Orozco-Aguilar J. Aguirre F. Tacchi F. Simon F. (2020). Role of oxidative stress in hepatic and extrahepatic dysfunctions during nonalcoholic fatty liver disease (NAFLD). Oxidative Med. Cell. Longev. 2020 (1), 1617805. 10.1155/2020/1617805 33149804 PMC7603619

[B26] Gulsen M. Yesilova Z. Bagci S. Uygun A. Ozcan A. Ercin C. N. (2005). Elevated plasma homocysteine concentrations as a predictor of steatohepatitis in patients with non‐alcoholic fatty liver disease. J. Gastroenterology Hepatology 20 (9), 1448–1455. 10.1111/j.1440-1746.2005.03891.x 16105135

[B27] Hao X. Ma C. Xiang T. Ou L. Zeng Q. (2021). Associations among methylene tetrahydrofolate reductase rs1801133 C677T gene variant, food groups, and non-alcoholic fatty liver disease risk in the Chinese population. Front. Genetics 12, 568398. 10.3389/fgene.2021.568398 33679874 PMC7930608

[B28] Harb Z. Deckert V. Bressenot A. M. Christov C. Gueant-Rodriguez R.-M. Raso J. (2020). The deficit in folate and vitamin B12 triggers liver macrovesicular steatosis and inflammation in rats with dextran sodium sulfate-induced colitis. J. Nutr. Biochem. 84, 108415. 10.1016/j.jnutbio.2020.108415 32645655

[B29] He Y.-M. Zhu S. Ge Y.-W. Kazuma K. Zou K. Cai S.-Q. (2015). The anti-inflammatory secoiridoid glycosides from Gentianae Scabrae Radix: the root and rhizome of Gentiana scabra. J. Nat. Med. 69 (3), 303–312. 10.1007/s11418-015-0894-8 25750086

[B30] Hegazy M. Saleh S. A. Ezzat A. Behiry M. E. (2020). Novel application of the traditional lipid ratios as strong risk predictors of NASH. Diabetes Metab. Syndr. Obes., 297–305. 10.2147/DMSO.S229590 PMC7021917 32104026

[B31] Higashi Y. (2023). Endothelial function in dyslipidemia: roles of LDL-cholesterol, HDL-Cholesterol and triglycerides. Cells 12 (9), 1293. 10.3390/cells12091293 37174693 PMC10177132

[B32] Huang Y.-S. Chang T.-E. Perng C.-L. Huang Y.-H. (2021). Genetic variations of three important antioxidative enzymes SOD2, CAT, and GPX1 in nonalcoholic steatohepatitis. J. Chin. Med. Assoc. 84 (1), 14–18. 10.1097/JCMA.0000000000000437 33009206 PMC12965991

[B33] Huang C. Yong Q. Lu Y. Wang L. Zheng Y. Zhao L. (2024). Gentiopicroside improves non-alcoholic steatohepatitis by activating PPARα and suppressing HIF1. Front. Pharmacology 15, 1335814. 10.3389/fphar.2024.1335814 38515850 PMC10956515

[B34] Ji X. Ma Q. Wang X. Ming H. Bao G. Fu M. (2023). Digeda-4 decoction and its disassembled prescriptions improve dyslipidemia and apoptosis by regulating AMPK/SIRT1 pathway on tyloxapol-induced nonalcoholic fatty liver disease in mice. J. Ethnopharmacol. 317, 116827. 10.1016/j.jep.2023.116827 37348794

[B35] Kadi D. Loomba R. Bashir M. R. (2024). Diagnosis and monitoring of nonalcoholic steatohepatitis: current state and future directions. Radiology 310 (1), e222695. 10.1148/radiol.222695 38226882

[B36] Kim S. Lindner S. N. Aslan S. Yishai O. Wenk S. Schann K. (2020). Growth of E. coli on formate and methanol *via* the reductive glycine pathway. Nat. Chemical Biology 16 (5), 538–545. 10.1038/s41589-020-0473-5 32042198

[B37] Koehler E. M. Schouten J. N. Hansen B. E. Hofman A. Stricker B. H. Janssen H. L. (2013). External validation of the fatty liver index for identifying nonalcoholic fatty liver disease in a population-based study. Clin. Gastroenterol. Hepatol. 11 (9), 1201–1204. 10.1016/j.cgh.2012.12.031 23353640

[B38] Kou B. Jiang Y. Chen Y. Yang J. Sun J. Yan Y. (2023). A Study of Gentianae Radix et Rhizoma Class Differences Based on Chemical Composition and Core Efficacy. Molecules 28 (20), 7132. 10.3390/molecules28207132 37894611 PMC10609378

[B39] Krushkal J. Zhao Y. Hose C. Monks A. Doroshow J. H. Simon R. (2016). Concerted changes in transcriptional regulation of genes involved in DNA methylation, demethylation, and folate-mediated one-carbon metabolism pathways in the NCI-60 cancer cell line panel in response to cancer drug treatment. Clin. Epigenetics 8 (1), 73. 10.1186/s13148-016-0240-3 27347216 PMC4919895

[B40] Kusnadi E. P. Timpone C. Topisirovic I. Larsson O. Furic L. (2022). Regulation of gene expression *via* translational buffering. Biochimica Biophysica Acta (BBA) - Mol. Cell Res. 1869 (1), 119140. 10.1016/j.bbamcr.2021.119140 34599983

[B41] Lai J. Wang H. L. Zhang X. Wang H. Liu X. (2022). Pathologic diagnosis of nonalcoholic fatty liver disease. Archives Pathology Laboratory Med. 146 (8), 940–946. 10.5858/arpa.2021-0339-RA 34871361

[B42] Lee K.-C. Wu P.-S. Lin H.-C. (2022). Pathogenesis and treatment of non-alcoholic steatohepatitis and its fibrosis. Clin. Molecular Hepatology 29 (1), 77–98. 10.3350/cmh.2022.0237 36226471 PMC9845678

[B43] Lei P. Cao L. Zhang H. Fu J. Wei X. Zhou F. (2024). Polyene phosphatidylcholine enhances the therapeutic response of oxaliplatin in gastric cancer through Nrf2/HMOX1 mediated ferroptosis. Transl. Oncol. 43, 101911. 10.1016/j.tranon.2024.101911 38377934 PMC10891348

[B44] Li R.-J. Ji W.-Q. Pang J.-J. Wang J.-L. Chen Y.-G. Zhang Y. (2013). Alpha-lipoic acid ameliorates oxidative stress by increasing aldehyde dehydrogenase-2 activity in patients with acute coronary syndrome. Tohoku Journal Experimental Medicine 229 (1), 45–51. 10.1620/tjem.229.45 23238616

[B45] Li X. Bu S. Pan R. R. Zhou C. Qu K. Ying X. (2020). The values of AHCY and CBS promoter methylation on the diagnosis of cerebral infarction in Chinese Han population. BMC Medical Genomics 13 (1), 163. 10.1186/s12920-020-00798-7 33138824 PMC7607831

[B46] Li Y. Chen A. Li Z. Cui X. Zhang G. (2022). Effectiveness of polyene phosphatidylcholine and its combination with other drugs in patients with liver diseases based on real-world research. Expert Review Clinical Pharmacology 15 (11), 1363–1375. 10.1080/17512433.2022.2121700 36062967

[B47] Li Z.-M. Kong C.-Y. Mao Y.-Q. Chen H.-L. Zhang S.-L. Huang J.-T. (2023). Host ALDH2 deficiency aggravates nonalcoholic steatohepatitis through gut-liver axis. Pharmacol. Res. 196, 106902. 10.1016/j.phrs.2023.106902 37657657

[B48] Liew S.-C. Gupta E. D. (2015). Methylenetetrahydrofolate reductase (MTHFR) C677T polymorphism: epidemiology, metabolism and the associated diseases. Eur. Journal Medical Genetics 58 (1), 1–10. 10.1016/j.ejmg.2014.10.004 25449138

[B49] Liu W. Baker S. S. D Baker R. Zhu L. (2015). Antioxidant mechanisms in nonalcoholic fatty liver disease. Curr. Drug Targets 16 (12), 1301–1314. 10.2174/1389450116666150427155342 25915484

[B50] Liu G.-K. Yang Q. Ye F.-Q. Niu Z. Zhang B.-Y. Kang N. (2024). Benzoate glycosides from Gentiana scabra bge. And their lipid-lowering activity. Phytochemistry 226, 114209. 10.1016/j.phytochem.2024.114209 38972439

[B51] Lu Y. Feng T. Zhao J. Jiang P. Xu D. Zhou M. (2022). Polyene phosphatidylcholine ameliorates high fat diet-induced non-alcoholic fatty liver disease *via* remodeling metabolism and inflammation. Front. Physiology 13, 810143. 10.3389/fphys.2022.810143 35295576 PMC8918669

[B52] Mairinoja L. Heikelä H. Blom S. Kumar D. Knuuttila A. Boyd S. (2023). Deep learning–based image analysis of liver steatosis in mouse models. Am. J. Pathology 193 (8), 1072–1080. 10.1016/j.ajpath.2023.04.014 37236505 PMC12178343

[B53] Martín-Fernández M. Arroyo V. Carnicero C. Sigüenza R. Busta R. Mora N. (2022). Role of oxidative stress and lipid peroxidation in the pathophysiology of NAFLD. Antioxidants 11 (11), 2217. 10.3390/antiox11112217 36358589 PMC9686676

[B54] Mato J. Martínez-Chantar M. Noureddin M. Lu S. (2017). “One-carbon metabolism in liver health and disease,” in Liver pathophysiology (Elsevier), 761–765.

[B55] Matthews R. G. Sheppard C. Goulding C. (1998). Methylenetetrahydrofolate reductase and methionine synthase: biochemistry and molecular biology. Eur. Journal Pediatrics 157 (Suppl. 2), S54–S59. 10.1007/pl00014305 9587027

[B56] Matthews R. P. Lorent K. Mañoral-Mobias R. Huang Y. Gong W. Murray I. V. (2009). TNFα-dependent hepatic steatosis and liver degeneration caused by mutation of zebrafish S-adenosylhomocysteine hydrolase. Development 136 (5), 865–875. 10.1242/dev.027565 19201949 PMC2685951

[B57] Morellato A. E. Umansky C. Pontel L. B. (2021). The toxic side of one-carbon metabolism and epigenetics. Redox Biol. 40, 101850. 10.1016/j.redox.2020.101850 33418141 PMC7804977

[B58] Murray B. Barbier-Torres L. Fan W. Mato J. M. Lu S. C. (2019). Methionine adenosyltransferases in liver cancer. World Journal Gastroenterology 25 (31), 4300–4319. 10.3748/wjg.v25.i31.4300 31496615 PMC6710175

[B59] Murthy S. N. Matta A. S. Mondal D. McNamara D. B. (2003). Methods in assessing homocysteine metabolism. Etabolic Syndr. Relat. Disord. 1 (2), 129–140. 10.1089/154041903322294452 18370634

[B60] Nattermann M. Wenk S. Pfister P. He H. Lee S. H. Szymanski W. (2023). Engineering a new-to-nature Cascade for phosphate-dependent formate to formaldehyde conversion *in vitro* and *in vivo* . Nat. Communications 14 (1), 2682. 10.1038/s41467-023-38072-w 37160875 PMC10170137

[B61] Pogribny I. P. Dreval K. Kindrat I. Melnyk S. Jimenez L. de Conti A. (2017). Epigenetically mediated inhibition of S-adenosylhomocysteine hydrolase and the associated dysregulation of 1-carbon metabolism in nonalcoholic steatohepatitis and hepatocellular carcinoma. FASEB J. 32 (3), 1591–1601. 10.1096/fj.201700866R 29127188 PMC6137451

[B62] Prysyazhnyuk V. Sydorchuk L. Sydorchuk R. Prysiazhniuk I. Bobkovych K. Buzdugan I. (2021). Glutathione-S-transferases genes-promising predictors of hepatic dysfunction. World Journal Hepatology 13 (6), 620–633. 10.4254/wjh.v13.i6.620 34239698 PMC8239493

[B63] Quinn C. Rico M. C. Merali C. Merali S. (2022). Dysregulation of S-adenosylmethionine metabolism in nonalcoholic steatohepatitis leads to polyamine flux and oxidative stress. Int. Journal Molecular Sciences 23 (4), 1986. 10.3390/ijms23041986 35216100 PMC8878801

[B64] Radziejewska A. Muzsik A. Milagro F. I. Martínez J. A. Chmurzynska A. (2020). One-carbon metabolism and nonalcoholic fatty liver disease: the crosstalk between nutrients, microbiota, and genetics. Lifestyle Genomics 13 (2), 53–63. 10.1159/000504602 31846961

[B65] Raghubeer S. Matsha T. E. (2021). Methylenetetrahydrofolate (MTHFR), the one-carbon cycle, and cardiovascular risks. Nutrients 13 (12), 4562. 10.3390/nu13124562 34960114 PMC8703276

[B66] Ramani K. Lu S. C. (2017). Methionine adenosyltransferases in liver health and diseases. Liver Research 1 (2), 103–111. 10.1016/j.livres.2017.07.002 29170720 PMC5695885

[B67] Ridpath J. R. Nakamura A. Tano K. Luke A. M. Sonoda E. Arakawa H. (2007). Cells deficient in the FANC/BRCA pathway are hypersensitive to plasma levels of formaldehyde. Cancer Research 67 (23), 11117–11122. 10.1158/0008-5472.CAN-07-3028 18056434

[B68] Riva G. Villanova M. Cima L. Ghimenton C. Bronzoni C. Colombari R. (2018). “Oil red O is a useful tool to assess donor liver steatosis on frozen sections during transplantation,” in Transplantation proceedings. 10.1016/j.transproceed.2018.06.013 30577233

[B69] Robert K. Nehmé J. Bourdon E. Pivert G. Friguet B. Delcayre C. (2005). Cystathionine β synthase deficiency promotes oxidative stress, fibrosis, and steatosis in mice liver. Gastroenterology 128 (5), 1405–1415. 10.1053/j.gastro.2005.02.034 15887121

[B70] Robinson A. E. Binek A. Ramani K. Sundararaman N. Barbier-Torres L. Murray B. (2023). Hyperphosphorylation of hepatic proteome characterizes nonalcoholic fatty liver disease in S-adenosylmethionine deficiency. Iscience 26 (2), 105987. 10.1016/j.isci.2023.105987 36756374 PMC9900401

[B71] Sáenz de Urturi D. Buqué X. Porteiro B. Folgueira C. Mora A. Delgado T. C. (2022). Methionine adenosyltransferase 1a antisense oligonucleotides activate the liver-brown adipose tissue axis preventing obesity and associated hepatosteatosis. Nat. Communications 13 (1), 1096. 10.1038/s41467-022-28749-z 35232994 PMC8888704

[B72] Schug Z. T. (2018). Formaldehyde detoxification creates a new wheel for the folate-driven one-carbon “bi”-cycle. Biochemistry, 57 (6), 889–890. 10.1021/acs.biochem.7b01261 29368500

[B73] Shao Y. Zhu H. Chen X. Feng E. Chen C. Shao Z. (2025). Associations of ALT, AST and ALT/AST ratio with metabolically unhealthy obesity in the elderly. Front. Nutr. 12, 1513029. 10.3389/fnut.2025.1513029 40196018 PMC11973076

[B74] Tang Y. Chen X. Chen Q. Xiao J. Mi J. Liu Q. (2022). Association of serum methionine metabolites with non-alcoholic fatty liver disease: a cross-sectional study. Nutr. and Metabolism 19 (1), 21. 10.1186/s12986-022-00647-7 35303918 PMC8932073

[B75] Tripathi M. Singh B. K. Zhou J. Tikno K. Widjaja A. Sandireddy R. (2022). Vitamin B12 and folate decrease inflammation and fibrosis in NASH by preventing syntaxin 17 homocysteinylation. J. Hepatology 77 (5), 1246–1255. 10.1016/j.jhep.2022.06.033 35820507

[B76] Umansky C. Morellato A. E. Rieckher M. Scheidegger M. A. Martinefski M. R. Fernández G. A. (2022). Endogenous formaldehyde scavenges cellular glutathione resulting in redox disruption and cytotoxicity. Nat. Communications 13 (1), 745. 10.1038/s41467-022-28242-7 35136057 PMC8827065

[B77] Vairetti M. Di Pasqua L. G. Cagna M. Richelmi P. Ferrigno A. Berardo C. (2021). Changes in glutathione content in liver diseases: an update. Antioxidants 10 (3), 364. 10.3390/antiox10030364 33670839 PMC7997318

[B78] Van der Crabben S. Wijburg F. Ackermans M. Sauerwein H. (2008). Effect of cysteine dosage on erythrocyte glutathione synthesis rate in a patient with cystathionine beta synthase deficiency. J. Inherit. Metabolic Dis. 31 (Suppl. 3), 469–475. 10.1007/s10545-007-0629-4 18213523

[B79] Vizán P. Di Croce L. Aranda S. (2021). Functional and pathological roles of AHCY. Front. Cell Developmental Biology 9, 654344. 10.3389/fcell.2021.654344 33869213 PMC8044520

[B80] Wang L. Jhee K.-H. Hua X. DiBello P. M. Jacobsen D. W. Kruger W. D. (2004). Modulation of cystathionine β-synthase level regulates total serum homocysteine in mice. Circulation Research 94 (10), 1318–1324. 10.1161/01.RES.0000129182.46440.4a 15105297

[B81] Wang Q. Zhou H. Bu Q. Wei S. Li L. Zhou J. (2022). Role of XBP1 in regulating the progression of non-alcoholic steatohepatitis. J. Hepatology 77 (2), 312–325. 10.1016/j.jhep.2022.02.031 35292349

[B82] Wei S. Wang L. Evans P. C. Xu S. (2024). NAFLD and NASH: etiology, targets and emerging therapies. Drug Discovery Today 29 (3), 103910. 10.1016/j.drudis.2024.103910 38301798

[B83] Werge M. P. McCann A. Galsgaard E. D. Holst D. Bugge A. Albrechtsen N. J. W. (2021). The role of the transsulfuration pathway in non-alcoholic fatty liver disease. J. Clinical Medicine 10 (5), 1081. 10.3390/jcm10051081 33807699 PMC7961611

[B84] Xiao H. Sun X. Lin Z. Yang Y. Zhang M. Xu Z. (2022). Gentiopicroside targets PAQR3 to activate the PI3K/AKT signaling pathway and ameliorate disordered glucose and lipid metabolism. Acta Pharm. Sin. B 12 (6), 2887–2904. 10.1016/j.apsb.2021.12.023 35755276 PMC9214054

[B85] Xu Z. Hao W. Xu D. He Y. Yan Z. Sun F. (2022). Polyene phosphatidylcholine interacting with TLR-2 prevents the synovial inflammation *via* inactivation of MAPK and NF-κB pathways. Inflammation 45 (4), 1507–1519. 10.1007/s10753-022-01633-0 35107766

[B86] Xuan Y. Wu D. Zhang Q. Yu Z. Yu J. Zhou D. (2024). Elevated ALT/AST ratio as a marker for NAFLD risk and severity: insights from a cross-sectional analysis in the United States. Front. Endocrinology 15, 1457598. 10.3389/fendo.2024.1457598 39253584 PMC11381241

[B87] Yang M. Qi X. Li N. Kaifi J. T. Chen S. Wheeler A. A. (2023). Western diet contributes to the pathogenesis of non-alcoholic steatohepatitis in Male mice *via* remodeling gut microbiota and increasing production of 2-oleoylglycerol. Nat. Communications 14 (1), 228. 10.1038/s41467-023-35861-1 36646715 PMC9842745

[B88] Yin Q. Song X. Yang P. Yang W. Li X. Wang X. (2023). Incorporation of glycyrrhizic acid and polyene phosphatidylcholine in lipid nanoparticles ameliorates acute liver injury *via* delivering p65 siRNA. Nanomedicine Nanotechnol. Biol. Med. 48, 102649. 10.1016/j.nano.2022.102649 36584740

[B89] Yong Q. Huang C. Chen B. An J. Zheng Y. Zhao L. (2024). Gentiopicroside improves NASH and liver fibrosis by suppressing TLR4 and NLRP3 signaling pathways. Biomed. Pharmacother. 177, 116952. 10.1016/j.biopha.2024.116952 38917754

[B90] Yu R. Lai Y. Hartwell H. J. Moeller B. C. Doyle-Eisele M. Kracko D. (2015). Formation, accumulation, and hydrolysis of endogenous and exogenous formaldehyde-induced DNA damage. Toxicol. Sciences 146 (1), 170–182. 10.1093/toxsci/kfv079 25904104 PMC4476463

[B91] Zeng Y. He H. An Z. (2022). Advance of serum biomarkers and combined diagnostic panels in nonalcoholic fatty liver disease. Dis. Markers 2022 (1), 1254014. 10.1155/2022/1254014 35811662 PMC9259243

[B92] Zhang C.-h. Xiao Q. Sheng J.-q. Liu T.-t. Cao Y.-q. Xue Y.-n. (2020). Gegen Qinlian Decoction abates nonalcoholic steatohepatitis associated liver injuries *via* anti-oxidative stress and anti-inflammatory response involved inhibition of toll-like receptor 4 signaling pathways. Biomed. Pharmacother. 126, 110076. 10.1016/j.biopha.2020.110076 32169759

[B93] Zubiete-Franco I. García-Rodríguez J. L. Martínez-Uña M. Martínez-Lopez N. Woodhoo A. Gutiérrez-De Juan V. (2016). Methionine and S-adenosylmethionine levels are critical regulators of PP2A activity modulating lipophagy during steatosis. J. Hepatology 64 (2), 409–418. 10.1016/j.jhep.2015.08.037 26394163 PMC4718902

