# Supplementary material for: Effect of gentiopicroside on endogenous formaldehyde homocysteine–pathway related proteins in rats with non-alcoholic steatohepatitis
Source: Front Pharmacol. 2026 Jan 6;16:1700101. doi: 10.3389/fphar.2025.1700101 (PMC12815877; doi:10.3389/fphar.2025.1700101)
Supplement: Supplementary file 1 [file Supplementaryfile1.docx]

Supplementary Material

# Supplementary Figures and Tables

## Supplementary Figures


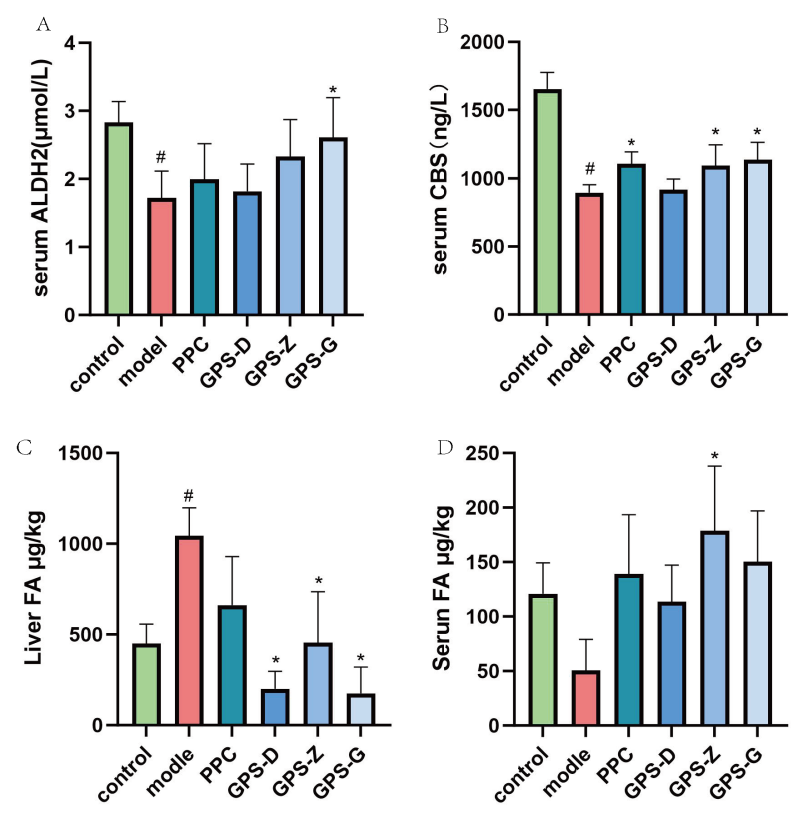


**Supplementary Figure 1. Expression levels of serum ALDH2, CBS, endogenous formaldehyde in the liver and serum of rats in each group**(A) Serum ALDH2 levels of rats in each group (n= 6); (B) Serum CBS levels of rats in each group (n= 6); (C) The levels of endogenous formaldehyde in the livers of rats in each group (n= 6); (D) The levels of endogenous formaldehyde in the serum of rats in each group (n= 6).

Note: Data are expressed as the mean ± SD. "#" indicates that *P*<0.05 compared with the normal group; "*" indicates that *P*<0.05 compared with the model group


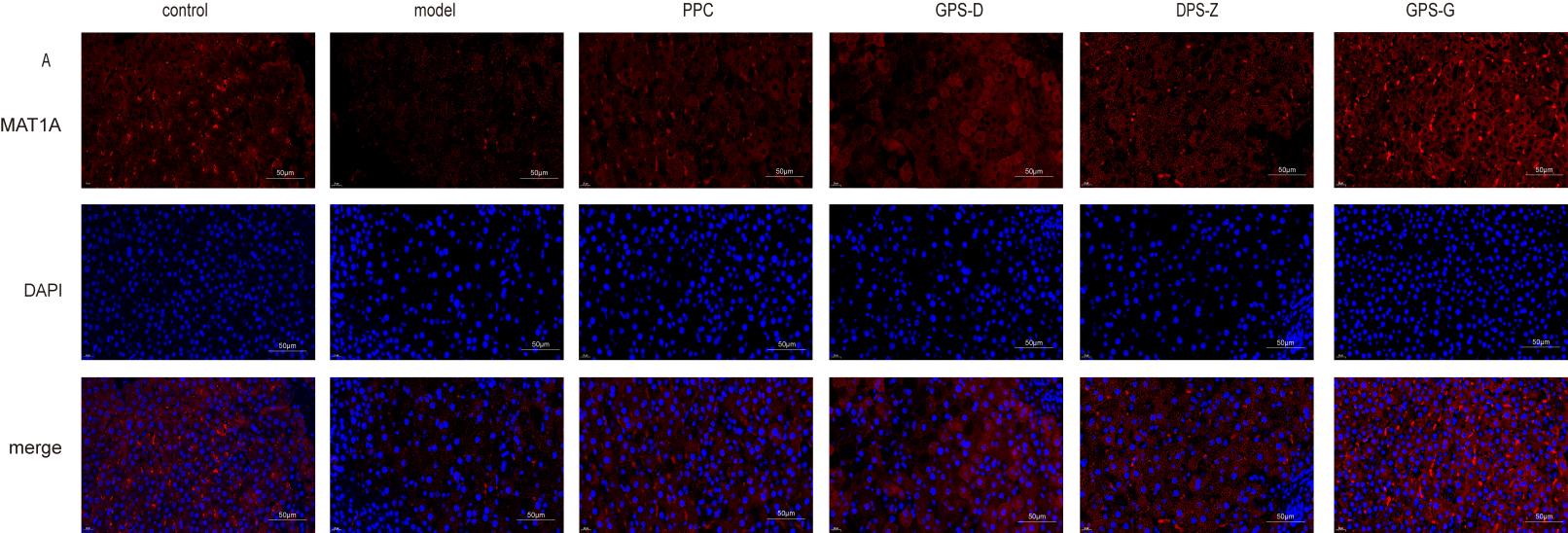


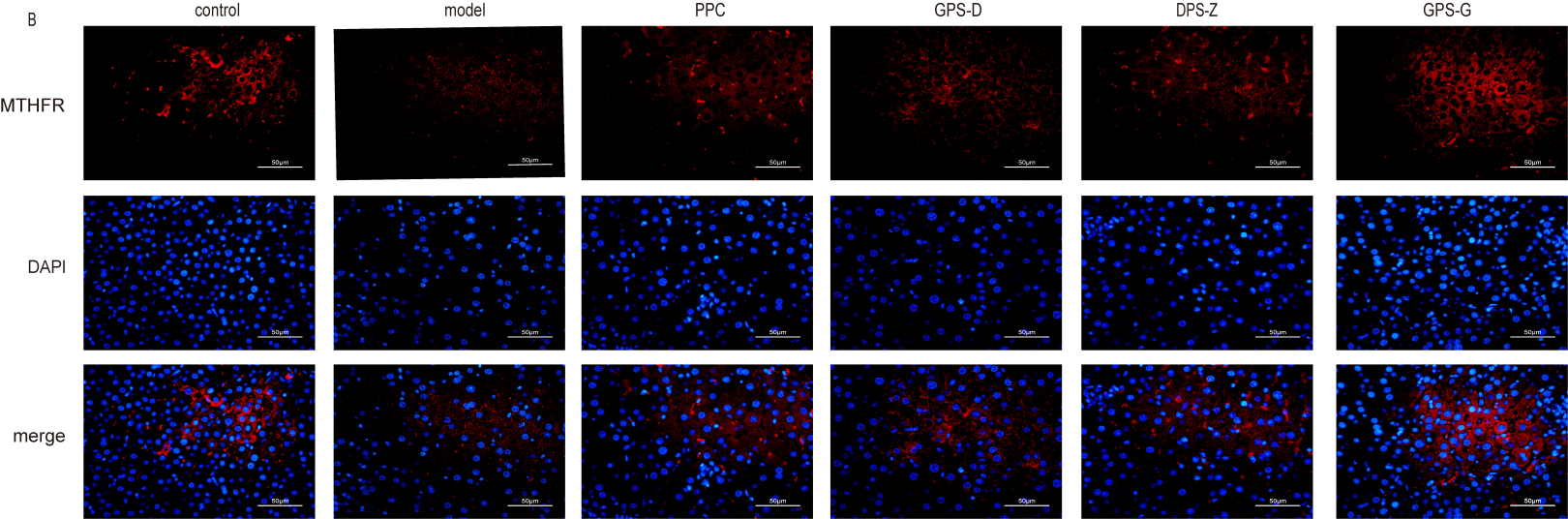


**Supplementary Figure 2.** (A) Fluorescence expression intensity of MAT1A in liver tissues of rats in each group; (B) Fluorescence expression intensity of MTHFR in liver tissues of rats in each group. Scale = 50μm
